# Safety and efficacy of combining midostaurin and gemtuzumab ozogamicin with induction chemotherapy in *FLT3*-mutated AML

**DOI:** 10.1182/bloodadvances.2025017244

**Published:** 2025-10-03

**Authors:** Nigel Russell, Jad Othman, Oliver Cumming, Abin Thomas, Aditya Tedjaseputra, Nicola Potter, Jelena Jovanovic, Amanda Gilkes, Leona Batten, Joanna Canham, Emily Hinson, Manohursingh Runglall, Phoebe Aucken, Panos Kottaridis, Jamie Cavenagh, Claire Arnold, Sylvie Freeman, Mike Dennis, Steven Knapper, Richard Dillon

**Affiliations:** 1 Department of Clinical Haematology, Guy’s and St Thomas’ Hospitals NHS Trust, London, United Kingdom; 2 Department of Medical and Molecular Genetics, King’s College, London, United Kingdom; 3 Faculty of Medicine and Health, The University of Sydney, Sydney, Australia; 4 Centre for Trials Research, Cardiff University, Cardiff, United Kingdom; 5 Department of Haematology, School of Medicine, Cardiff University, Cardiff, United Kingdom; 6 Department of Haematology, University College London Hospitals NHS Trust, London, United Kingdom; 7 Department of Haematology, Bart’s and the London Hospitals NHS Trust, London, United Kingdom; 8 Department of Haematology, Belfast City Hospital, Belfast, United Kingdom; 9 Clinical Immunology Service, College of Medicine and Health, University of Birmingham, Birmingham, United Kingdom; 10 Department of Haematology, The Christie Hospital NHS Trust, Manchester, United Kingdom

## Abstract

Despite the use of FMS-like tyrosine kinase 3 (FLT3) inhibitors, outcomes for patients with *FLT3*-mutated (*FLT3*^mut^) acute myeloid leukemia (AML) remain suboptimal because of high rates of relapse. We evaluated the safety and efficacy of the combination of daunorubicin, cytarabine (DA), gemtuzumab ozogamicin (GO), and midostaurin (DAGO+m) for younger patients with newly diagnosed *FLT3*^mut^ AML in the UK National Cancer Research Institute AML19 trial. A total of 195 patients were randomized to receive DA with either 1 or 2 doses of GO (DAGO1 and DAGO2). Overall, 77 had an *FLT3* mutation and received midostaurin for 2 weeks after each chemotherapy course and then as maintenance for 1 year unless they received a transplant. A total of 39 patients received DAGO1+m and 38 DAGO2+m. Their median age was 51 years (range, 20-74), and 16 (20%) were aged >60 years. The overall response rate was 91%. Day 60 mortality was 0%, with no increase in toxicity compared with patients treated contemporaneously with DAGO1 and DAGO2 without midostaurin. Two-year overall survival was 77%. Two-year event-free survival and cumulative incidence of relapse were 62% and 31%, respectively. Measurable residual disease (MRD) clearance was enhanced compared with patients with *FLT3*^mut^ AML treated with DAGO without midostaurin. Overall, 81% of evaluable patients were *NPM1* MRD negative in the peripheral blood after course 2 (76% with DAGO1+m, and 86% with DAGO2+m), 79% were MRD negative in the bone marrow by *FLT3*-ITD next-generation sequencing, and all patients had *FLT3*-MRD levels <0.01%. DAGO+m appears safe and effective. DAGO2+m will now be evaluated in a randomized study. This trial was registered at www.isrctn.com as #ISRCTN78449203.

## Key Points

• Midostaurin can be safely combined with GO and intensive induction chemotherapy in *FLT3*^mut^ AML.
• The addition of midostaurin to DAGO increased the clearance of molecular MRD assessed by RT-qPCR (for *NPM1* mutation) and NGS (for *FLT3*-ITD).

## Visual Abstract

**Figure:**
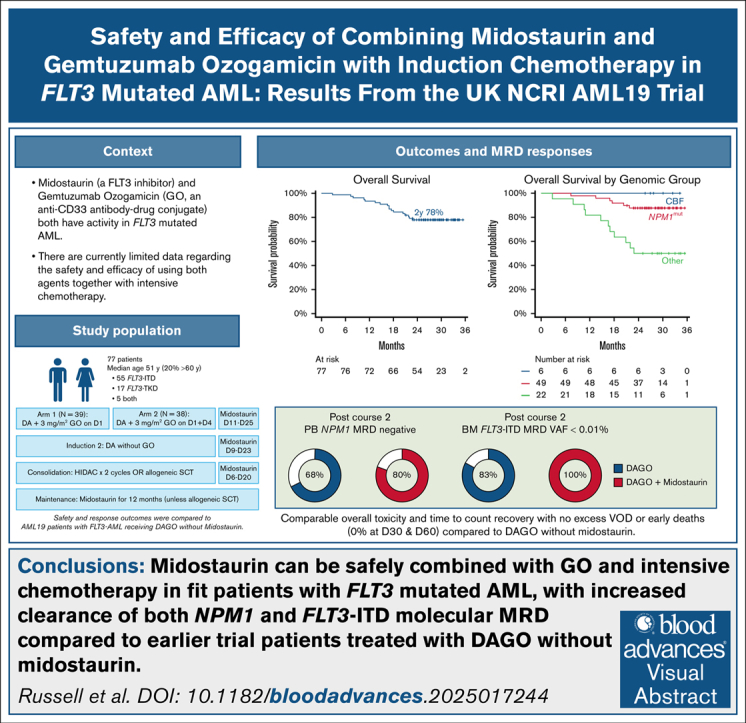

## Introduction

Activating somatic mutations in the gene encoding FMS-like tyrosine kinase 3 (*FLT3*) are present in approximately one-third of patients with acute myeloid leukemia (AML). Despite the incorporation of FLT3 inhibitors alongside intensive chemotherapy, outcomes remain suboptimal: 4-year overall survival (OS) was 51% in patients treated with daunorubicin, cytarabine (DA) and midostaurin in the RATIFY study^1^ and 48% in patients treated with DA and quizartinib in the QUANTUM-First study.^2^ In both, relapse was the leading cause of treatment failure, occurring in 42% and 34% of patients respectively.^2^^,^^3^ Therefore, improved treatment strategies are still clearly needed.

Gemtuzumab ozogamicin (GO) has been shown to improve survival in patients with favorable and intermediate risk cytogenetics, although the original studies did not evaluate *FLT3* mutation status.^4^ Nevertheless, blasts from patients with *FLT3*-mutated (*FLT3*^mut^) AML express high levels of CD33,^5^ and subgroup analyses from randomized studies suggest that patients with *FLT3*^mut^ AML benefited from the addition of GO to induction chemotherapy.^6^^,^^7^

Despite this, GO and FLT3 inhibitors are not routinely used together with intensive chemotherapy because of limited data regarding the safety and efficacy of these combinations.

To address this issue, we evaluated the combination of DA plus midostaurin with either 1 or 2 doses of GO within the UK National Cancer Research Institute (NCRI) AML19 trial. The aims were to evaluate safety and efficacy in terms of OS, event-free survival (EFS), and clearance of measurable residual disease (MRD).

## Methods

### Trial design and treatments

The NCRI AML19 trial (www.isrctn.com identifier: ISRCTN78449203) enrolled younger adults generally aged <60 years with newly diagnosed AML between November 2015 and November 2021. Older patients could enter if judged fit and after discussion with a trial coordinator. The results of the primary randomizations, in which no FLT3 inhibitors were used, have already been reported elsewhere.^8^^,^^9^

In November 2020, we amended the protocol to evaluate the combination of GO and midostaurin alongside DA chemotherapy (called AML19 version 2 [AML19v2]). From November 2020 to November 2021 we enrolled only patients without known adverse karyotype, and they were randomized 1:1 to receive induction chemotherapy with DA 3+10 (daunorubicin 60 mg/m^2^ on days 1, 3, and 5, and cytarabine 100 mg/m^2^ twice a day on days 1-10) with either a single dose of GO (3 mg/m^2^ on day 1 [DAGO1]) or 2 doses (3 mg/m^2^ capped at 5 mg on days 1 and 4 [DAGO2]). Patients underwent rapid centralized screening for *FLT3* mutations and if these were detected, patients were offered entry into a substudy (called Midotarg); those who consented received midostaurin for 14 days from day 11. After blood count recovery, the bone marrow (BM) was assessed, and if this showed complete remission (CR) or partial remission, they then received a second induction (DA 3+8, daunorubicin 50 mg/m^2^ on days 1, 3, and 5, plus cytarabine 100 mg/m^2^ twice daily on days 1-8) without GO and with midostaurin given for 14 days from day 9. Consolidation therapy was 2 courses of high-dose cytarabine (3 g/m^2^ twice daily on days 1, 3, and 5; reduced to 1.5 g/m^2^ in patients aged >60 years) with midostaurin for 14 days from day 6, followed by midostaurin maintenance for 1 year, except in patients proceeding to allogeneic stem cell transplant (allo-SCT). Patients without an *FLT3* mutation or who did not consent to the Midotarg substudy received the same chemotherapy but without midostaurin. Patients with refractory disease after course 1 (>15% blasts and <50% reduction in blasts) were recommended for salvage therapy with FLAG-Ida (fludarabine, cytarabine, and granulocyte colony-stimulating factor, and idarubicin), without midostaurin, as course 2. The trial schema is shown in supplemental Figure 1.

Patients could receive allo-SCT at any time at the discretion of the treating team, however allo-SCT in first CR (CR1) was recommended for patients with an *FLT3*-ITD allelic ratio (AR) of >0.05 without *NPM1* mutation or core binding factor (CBF) translocation, for patients with *NPM1* mutation who were MRD positive by reverse transcription quantitative polymerase chain reaction (RT-qPCR) in the peripheral blood (PB) after course 2 and for patients found to have adverse-risk cytogenetics after trial entry or who failed to achieve a CR/CR with incomplete hematological recovery (CRi) after 2 courses of induction.^10^ There was no protocol-specified posttransplant maintenance therapy. The primary end point was OS. Secondary end points included response (CR and CRi), MRD response, and toxicity (hematological and nonhematological).

Written informed consent was required for trial entry and for entry into the Midotarg substudy. The trial was approved by the Wales multicenter research ethics committee 3 (14/WA/1056) and conducted in accordance with the Declaration of Helsinki.

### Molecular and cytogenetic testing

Patients underwent screening for *FLT3* and *NPM1* mutations in a central laboratory. Cytogenetic testing was performed in accredited regional laboratories and reviewed centrally according to the Medical Research Council cytogenetic classification.^11^ RNA sequencing using a targeted panel (TruSight RNA Fusion, Illumina, Cambridge, United Kingdom) was performed centrally for patients with *FLT3*-ITD without an *NPM1* mutation or common fusion gene, and for patients in whom fusion genes were identified, these were confirmed by PCR. Testing for *UBTF* tandem duplication (*UBTF*-TD) was performed retrospectively by PCR, as previously described.^12^

### MRD

Molecular MRD assessment was performed prospectively for patients with *NPM1* mutations or fusion genes using RT-qPCR at a central reference laboratory, as previously described.^13^ Assessments were performed after each course of therapy and then every 3 months for 2 years with investigators informed of the results. Additional treatment was recommended for patients with MRD relapse according to the European Leukemia Network definitions,^14^ but was not protocol specified.

*FLT3*-ITD MRD was assessed retrospectively by next-generation sequencing using a modified getITD assay, as previously described.^15^^,^^16^ Briefly, this assay uses 500 ng genomic DNA and has a sensitivity of 0.001%.

To assess the effect of adding midostaurin, we compared MRD measurements against those from patients with the same genotype treated with DAGO without midostaurin in the first part of AML19 before the protocol amendment (AML19v1). Further details of the MRD testing and analysis methods are provided in the supplemental Appendix.

### Enhanced safety monitoring

In addition to data on serious adverse events (SAEs), we collected toxicity data for liver, kidney, and cardiac AEs (at any grade), and bleeding events (at grade 3 or 4) on a weekly basis for 4 weeks after the first dose of midostaurin for patients joining the Midotarg substudy, and this was reviewed by the trial team and by the independent data monitoring committee after 25 and 50 patients had been treated. The rate of SAEs was compared with that seen in contemporaneous patients not entering the substudy, who received the same induction therapy without midostaurin.

### Statistical analysis

Response end points were defined according to the revised International Working Group criteria.^17^ EFS was measured in all patients and was defined as time from randomization to treatment failure (refractory disease or partial response by the end of course 2, morphological or MRD relapse, or death from any cause). If treatment failure was due to refractory disease or partial response, the event was recorded on cycle 1 day 1. OS was defined as the time from randomization to death from any cause, with those still alive censored at the date last seen. Relapse-free survival was calculated only for patients who achieved CR or CRi, and was measured from the date of CR/CRi until the date of relapse (molecular or hematological) or death from any cause. Cumulative incidence of relapse, including molecular relapse, was calculated using cumulative incidence functions with nonrelapse mortality as a competing risk. Primary analyses were by intention to treat, and the final data cutoff was in January 2024. Survival outcomes were compared using Cox regression. Competing risk analysis was performed for the cumulative incidence of relapse with nonrelapse mortality as the competing risk, using the Gray test and the Fine and Gray model. Median follow-up was determined by reversing the censor indicator of Kaplan-Meier analysis for OS.

## Results

### Patients

From November 2020 to November 2021, 195 patients were randomized, 97 to DAGO1 and 98 to DAGO2. *FLT3* mutations were detected in 80 patients, of whom 77 consented to enter the Midotarg substudy and were allocated to receive midostaurin (39 DAGO1 plus midostaurin [DAGO1+m], and 38 DAGO2 plus midostaurin [DAGO2+m]). Of these, 55 had *FLT3*-ITD, 17 *FLT3–*tyrosine kinase domain, and 5 had both mutations. The baseline characteristics of those patients in the Midotarg substudy and those who received DAGO1 or DAGO2 without midostaurin are shown in Table 1.

**Table 1.** **Demographics of patients enrolled in AML19v2 trial, including those in the Midotarg substudy receiving DAGO+m and those receiving DAGO alone**

	Midotarg substudy			Other patients enrolled in AML19v2		
	Total	DAGO1+m	DAGO2+m	Total	DAGO1	DAGO2
n	77	39	38	118	58	60
Median age (range), y	51 (20-74)	52 (21-74)	50 (20-72)	50 (17-65)	48 (18-64)	50 (17-65)
**Age group, y**						
<30	5 (6.5)	2 (5.1)	3 (7.9)	14 (11.9)	8 (13.8)	6 (10.0)
30-39	13 (16.9)	6 (15.4)	7 (18.4)	17 (14.4)	9 (15.5)	8 (13.3)
40-49	19 (24.7)	10 (25.6)	9 (23.7)	25 (21.2)	11 (19.0)	14 (23.3)
50-59	24 (31.2)	13 (33.3)	11 (28.9)	49 (41.5)	23 (39.7)	26 (43.3)
≥60	16 (20.8)	8 (20.5)	8 (21.1)	13 (11)	7 (12.1)	6 (10.0)
**Gender**						
Male	37 (48.1)	19 (48.7)	18 (47.4)	58 (49.2)	29 (50.0)	29 (48.3)
Female	40 (51.9)	20 (51.3)	20 (52.6)	60 (50.8)	29 (50.0)	31 (51.7)
Previous hematological disorder	1 (1.3)	1 (2.6)	0 (0.0)	0 (0.0)	0 (0.0)	0 (0.0)
Previous chemotherapy or radiotherapy	1 (1.3)	1 (2.6)	0 (0.0)	3 (2.6)	2 (3.6)	1 (1.7)
**WBC count, ×10^9^/L**						
<10	27 (35.1)	14 (35.9)	13 (34.2)	60 (50.8)	28 (48.3)	32 (53.3)
10 to <50	36 (46.8)	18 (46.2)	18 (47.4)	47 (39.8)	24 (41.4)	23 (38.3)
50 to <100	7 (9.1)	4 (10.3)	3 (7.9)	9 (7.6)	5 (8.6)	4 (6.7)
≥100	7 (9.1)	3 (7.7)	4 (10.5)	2 (1.7)	1 (1.7)	1 (1.7)
**WHO performance status**						
Normal activity	36 (46.8)	18 (46.2)	18 (47.4)	68 (57.6)	34 (58.6)	34 (56.7)
Restricted activity	37 (48.1)	19 (48.7)	18 (47.4)	43 (36.4)	21 (36.2)	22 (36.7)
In bed <50% waking hours	4 (5.2)	2 (5.1)	2 (5.3)	7 (5.9)	3 (5.2)	4 (6.7)
*FLT3*-ITD mutation	60 (77.9)	29 (74.4)	31 (81.6)	3 (2.6)	2 (3.5)	1 (1.7)
*FLT3*-ITD AR, median (IQR)	0.46 (0.16-0.74)	0.45 (0.13-0.71)	0.48 (0.23-0.78)	0.70 (0.36-0.735)	0.74 (0.70-0.77)	0.02 (0.02-0.02)
*FLT3*-TKD mutation	22 (28.6)	12 (30.8)	10 (26.3)	0 (0.0)	0 (0.0)	0 (0.0)
*NPM1* mutation	49 (63.6)	25 (64.1)	24 (63.2)	32 (27.4)	16 (28.1)	16 (26.7)
**Cytogenetics (Grimwade et al****^11^****)**						
CBF	6 (7.8)	2 (5.1)	4 (10.5)	21 (17.8)	11 (19.0)	10 (16.7)
Normal	41 (53.2)	18 (46.2)	23 (60.5)	53 (44.9)	30 (51.7)	23 (38.3)
Other intermediate	23 (29.9)	15 (38.5)	8 (21.1)	23 (19.5)	10 (17.2)	13 (21.7)
Adverse	1 (1.3)	1 (2.6)	0 (0.0)	17 (14.4)	7 (12.1)	10 (16.7)
Failed	6 (7.8)	3 (7.7)	3 (7.9)	4 (3.3)	0 (0.0)	4 (6.7)

Data are presented as n (%) unless otherwise specified.

IQR, interquartile range; TKD, tyrosine kinase domain; WBC, white blood cell.

**Figure 1.**
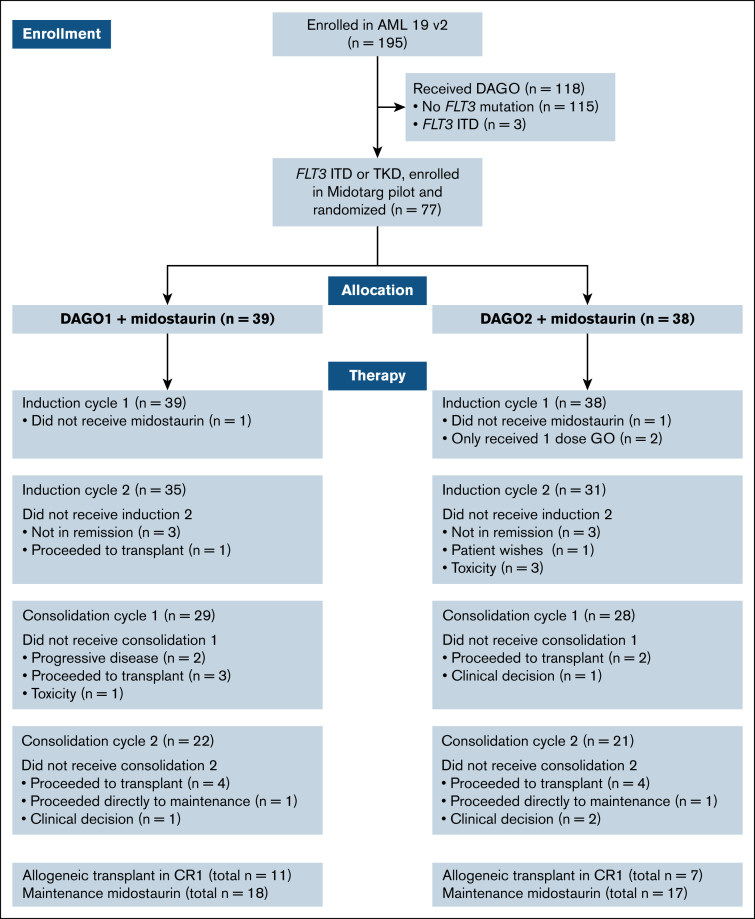
**CONSORT diagram showing the number of patients randomized, screened for *FLT3* mutations, and treated with and without midostaurin in each arm.** TKD, tyrosine kinase domain.

Of 77 patients who were allocated to receive midostaurin, the median age was 51 years with 16 (21%) aged >60 years. After the first course, 66 of 77 patients received DA as course 2, and 57 of 77 received at least 1 course of high-dose cytarabine consolidation. After the second course, 8 of 77 patients were designated high risk on the basis of *NPM1* MRD positivity in the PB. Overall, 18 of 77 patients (23%) received CR1 allo-SCT, of whom 11 had received DAGO1+m and 7 DAGO2+m. A CONSORT diagram is shown in Figure 1.

### Molecular and cytogenetic characteristics

*NPM1* mutations were present in 49 of 77 patients (64%). Medical Research Council cytogenetic risk group was favorable in 6 patients, intermediate in 64 (including 1 *KMT2A*::*MLLT3* and 1 *DEK*::*NUP214*), unknown in 6, and adverse in 1 patient. Of 20 patients without *NPM1* mutation or a common fusion gene, 16 patients underwent RNA sequencing, revealing *KMT2A* partial tandem duplication in 5 patients and *ETV6*::*MECOM* fusion in 1 patient. *UBTF*-TD was detected in 5 patients.

### Compliance and toxicity

Of 77 patients, 64 (83%) received all 28 doses of prescribed midostaurin in course 1 or missed no more than 1 dose, the remaining patients missed between 3 and 20 doses. Two patients randomized to DAGO1+m did not receive midostaurin in course 1 due to gastrointestinal intolerance, and 1 patient randomized to DAGO2+m did not receive midostaurin due to a preexisting QTc prolongation. Seventeen SAEs (grade ≥3) were reported (DAGO1+m, n = 11; DAGO2+m, n = 6). No cases of veno-occlusive disorder (VOD) were reported.

We could not find evidence that blood count recovery was delayed in patients receiving midostaurin compared with those receiving DAGO alone. In course 1, time to neutrophil recovery to ≥1 × 10^9^/L was 33 and 33 days with DAGO1+m and DAGO2+m, respectively, compared with 32 and 32 days in patients receiving DAGO1 and DAGO2 alone, respectively, without midostaurin in AML19v2 (Table 2). Likewise, time to platelet recovery to ≥100 × 10^9^/L was not delayed with DAGO+m compared with DAGO alone (Table 2).

**Table 2.** **Count recovery times and resource usage**

	DAGO1	DAGO1+m	*P* value	DAGO2	DAGO2+m	*P* value
**Recovery times**						
Neutrophil recovery to 1 × 10^9^/L, median (95% CI), d	32 (28-34)	33 (28-36)	.37	32 (30-35)	33 (30-38)	.40
Platelet recovery to 100 × 10^9^/L, median (95% CI), d	28 (26-30)	27 (26-30)	.814	29 (27-30)	28 (27-32)	.580
**Resource use, median (IQR)**						
Units of blood	8 (6-13)	10 (7-11)	.445	9 (7-12)	7 (6-9)	.029
Units of platelets	10 (6.5-14)	11.5 (8-15)	.406	13 (10-17)	9 (6.5-12)	.001
Days of IV antibiotics	17 (13-27.5)	17 (12-26)	.804	21 (15-28)	20 (12-25)	.147
Nights in hospital	36 (30-40)	39 (31-50)	.103	36 (31-40)	36 (28-43)	.884

**Figure 2.**
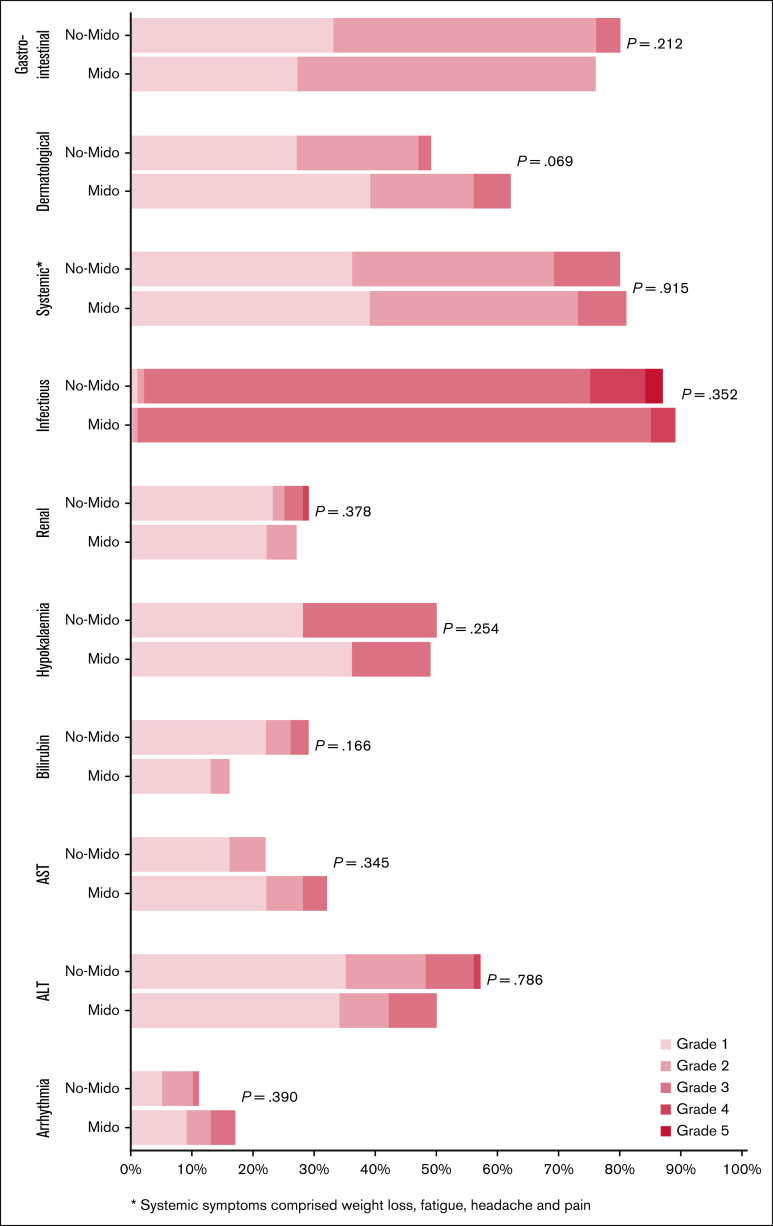
**Nonhematological toxicity of patients entering the Midotarg substudy, compared with patients in AML19v2 who did not enter and were treated with DAGO without midostaurin.** ALT, alanine aminotransferase; AST, aspartate aminotransferase; Mido, midostaurin.

There was no significant difference in nonhematological toxicity between patients who did and did not receive midostaurin in AML19v2 (Figure 2), nor was there a significant difference in nonhematological toxicity between DAGO1+m and DAGO2+m (supplemental Figure 2). Day-30 and day-60 mortality for both DAGO1+m and DAGO2+m was 0%.

Midostaurin maintenance was administered in 18 of 39 patients (46%) randomized to DAGO1+m, and 17 of 38 (45%) randomized to DAGO2+m, with a median of 12 cycles administered in both groups (Figure 1).

### Response

The overall response rate (including CR and CRi) after the first course of induction was 87%, 85% for DAGO1+m and 89% for DAGO2+m. After 2 courses of induction, CR/CRi was achieved in 91% and did not differ between DAGO1+m (90%) and DAGO2+m (92%; Table 3). There were 10 patients not in remission after course 1, 6 in the DAGO1+m and 4 in the DAGO2+m arm. Of these, 4 had achieved partial remission (<15% blasts in the BM) and received course 2 of DA+m as per protocol. The 6 patients with refractory disease after course 1 were treated off protocol with FLAG-Ida (n = 3), gilteritinib (n = 2), or azacytidine (n = 1).

**Table 3.** **Outcomes for patients enrolled in the Midotarg substudy receiving DAGO+m**

	DAGO1+m n = 39	DAGO2+m n = 38
**Response, %**		
ORR (CR + CRi)	90	92
CR	77	81.5
CRi	13	10.5
CR/CR_i_ after course 1	85	89
Postcourse 2 PB MRD negative for *NPM1*	18/24 (75%)	19/22 (86%)
Allogeneic transplant in CR1	11	7
**Survival, %**		
2-Year OS	76	78
2-Year EFS	59	66
2-Year cumulative incidence of relapse	33	29
2-Year relapse-free survival	66	71

ORR, overall response rate.

### MRD

**Figure 3.**
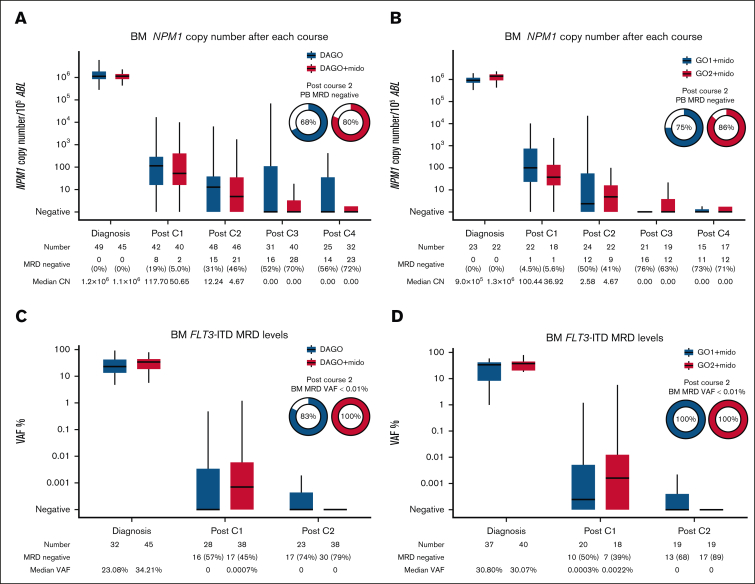
**MRD as assessed in the BM.** (A) *NPM1* MRD measured by quantitative reverse transcription PCR after each treatment cycle. Patients with *NPM1* and *FLT3* mutations treated with DAGO (n = 55) without midostaurin in AML19v1 are shown for comparison. (B) *NPM1* MRD by GO dose for patients in the Midotarg substudy. (C) *FLT3*-ITD next-generation sequencing (NGS) MRD after treatment cycles 1 and 2. Patients with *FLT3*-ITD mutations treated with DAGO without midostaurin in AML19v1 are shown for comparison. (D) *FLT3*-ITD NGS MRD by GO dose for patients in Midotarg substudy. CN, copy number; Mido, midostaurin; VAF, variant allele frequency.

BM MRD levels after each course of chemotherapy for patients with *NPM1* mutation are shown in Figure 3A. For comparison, we identified 55 patients with both *NPM1* and *FLT3* mutations treated with DAGO1 and DAGO2 without midostaurin in the preceding AML19v1 protocol. The characteristics of the patients are shown in supplemental Table 1. The 2 groups were generally comparable although there was a higher proportion of patients with *FLT3*-ITD and a higher AR in the midostaurin-treated group. BM *NPM1* MRD levels at the end of treatment (after course 4) were lower in patients receiving midostaurin: 72% became MRD negative with DAGO+m compared with 56% for DAGO without midostaurin in AML19v1.

We previously showed that PB *NPM1* MRD status after course 2 (PC2) provides more powerful prognostic information than BM,^10^ and PC2 PB *NPM1* MRD status was used to allocate CR1 allo-SCT in this study. PC2 PB MRD negativity was 80% in patients receiving DAGO+m compared with 68% in those receiving DAGO without midostaurin in AML19v1.

Next, we analyzed the effect of GO dose on MRD levels in patients receiving midostaurin. More patients receiving DAGO2+m were PB MRD negative after course 2 (75% and 86% for DAGO1+m and DAGO2+m, respectively; Figure 3B; Table 3). For patients receiving DAGO1 and DAGO2 without midostaurin in AML19v1 these figures were 61% and 74%, respectively.

Next-generation sequencing was used to detect *FLT3*-ITD MRD (Figure 3C-D; supplemental Figure 3). For comparison, we selected 32 patients with *FLT3*-ITD treated with DAGO without midostaurin in AML19v1 who had available samples. The characteristics of all patients in the *FLT3*-ITD MRD analysis are shown in supplemental Table 2. *FLT3*-ITD MRD negativity in the BM after cycle 2 was attained in 30 of 38 patients (79%) treated with midostaurin, this was 68% and 89% in those patients treated with DAGO1+m and DAGO2+m, respectively, and no patient had an *FLT3*-ITD variant allele frequency of ≥0.01%.

Although the rates of BM *FLT3* MRD negativity were similar in patients treated without midostaurin (73% and 75% for DAGO1 and DAGO2, respectively), more of these patients were MRD positive above a level of 0.01% (17% vs 0% for DAGO vs DAGO+m; *P* = .017).

### Survival outcomes

**Figure 4.**
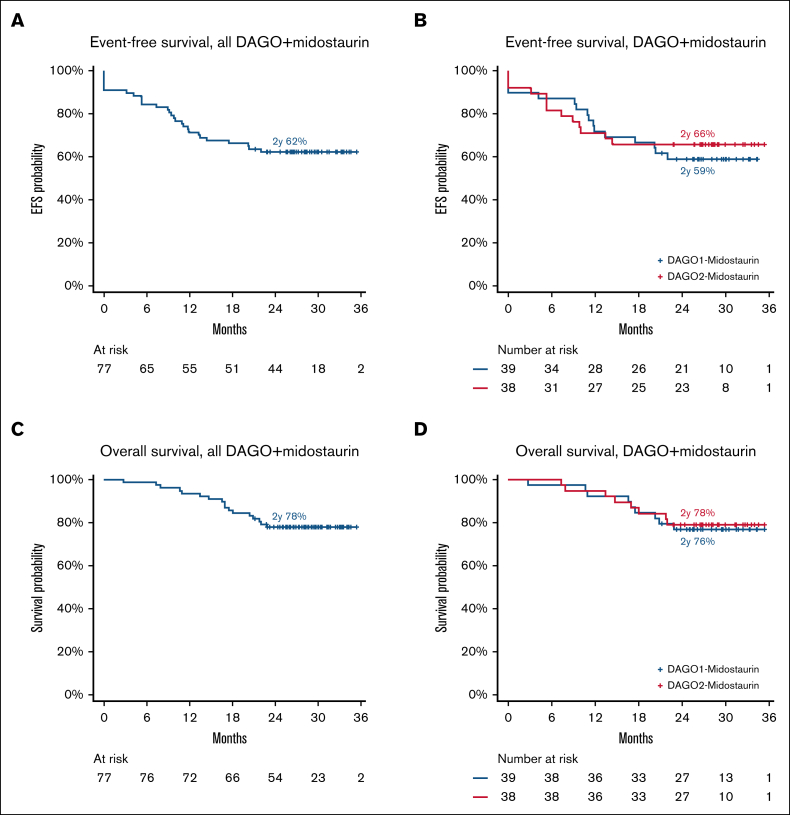
**Survival outcomes for patients in AML19v2.** (A) EFS for all patients in the Midotarg substudy. (B) EFS by randomization (DAGO1+m vs DAGO2+m). (C) OS for all patients in the Midotarg substudy. (D) OS by GO randomization (DAGO1+m vs DAGO2+m).

With a median follow-up of 28.9 months, 2-year EFS and OS among all patients in the Midotarg substudy was 62% and 78%, respectively (Figure 4A-B) and did not differ between DAGO1+m and DAGO2+m (Figure 4C-D; Table 3). EFS at 2 years was 59% and 66% for DAGO1+m and DAGO2+m, respectively (hazard ratio [HR], 0.86; 95% confidence interval [CI], 0.41-1.79; *P* = .68). Likewise, there was no difference in OS (HR, 0.90; 95% CI, 0.35-2.35; *P* = .83), which, at 2 years, was 76% for DAGO1+m and 78% for DAGO2+m (Figure 4). The cumulative incidence of relapse was 31% at 2 years and did not vary by GO dose (HR, 0.94; 95% CI, 0.40-2.20; *P* = .88; supplemental Figure 4A-B). Likewise, there was no difference in relapse-free survival by GO dose (HR, 0.88; 95% CI, 0.38-2.04; *P* = .77), which was 66% vs 71% for DAGO1+m and DAGO2+m, respectively (supplemental Figure 4C-D). For patients who received a transplant in first remission (n = 18) OS at 2 years was 73% and 100% for DAGO1+m and DAGO2+m, respectively (supplemental Figure 5).

### Exploratory analyses of clinical and molecular subgroups

**Figure 5.**
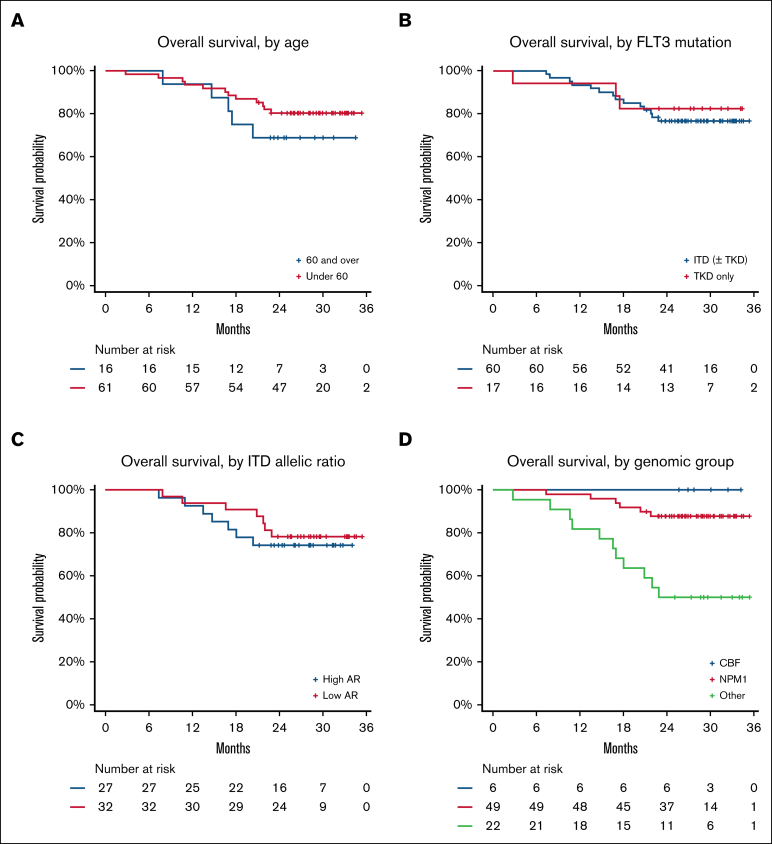
**Exploratory analyses of OS in clinical and molecular subgroups.** (A) According to age (>60 years or <60 years). (B) According to *FLT3* mutation type. (C) According to AR for patients with *FLT3*-ITD mutation. (D) According to genomic subgroup (CBF AML, *NPM1*^mut^ AML, and others). TKD, tyrosine kinase domain.

Age did not significantly affect survival. In the 16 patients aged >60 years, 2-year OS was 69% compared with 80% in younger patients (*P* = .6; Figure 5A). There was no difference in EFS or OS between patients with *FLT3*-ITD and those with *FLT3*–tyrosine kinase domain or by *FLT3* AR (Figure 5B-C). In contrast, there were major differences in survival among different genomic groups. Patients with *NPM1* mutation and CBF AML had excellent outcomes, with 2-year OS of 88% and 100%, respectively; patients without either of these lesions (n = 22) had poorer survival (*P* < .001; Figure 5D). This group included patients with *UBTF*-TD (n = 5), *KMT2A*::*MLLT3* (n = 1), *DEK*::*NUP214* (n = 1), *ETV6*::*MECOM* (n = 1), monosomy 7 (n = 1), and *KMT2A* partial TD (n = 3), as well as 6 patients with normal, and 4 patients with other intermediate, karyotypes who could not be further subclassified.

## Discussion

Previous studies have shown that both a single-dose and fractionated dosing schedule of GO can safely be combined with DA chemotherapy in adult patients fit for intensive therapy including older patients.^8^^,^^18^, ^19^, ^20^ We now show that midostaurin can safely be added to that combination. The triplet was generally well tolerated in both younger and older patients, with no day-60 mortality and no increase in hematological or nonhematological toxicity compared with patients receiving the same chemotherapy without midostaurin. Compliance was good and >80% of patients were able to complete midostaurin in course 1. Furthermore, there was no additional toxicity with DAGO2+m compared with DAGO1+m. The safety of the DAGO+m combination is supported by a number other smaller studies. The Study Alliance Leukemia cooperative group recently reported a phase 1 study^21^ (MOSAIC) of 11 patients combining DAGO with midostaurin, confirming the safety and feasibility of delivering the triplet using a fractionated GO schedule (3 mg/m^2^ on days 1 and 4). A Czech AML group study also reported on 11 patients, combing midostaurin with a fractionated 3-dose schedule of GO (3 mg/m^2^ on days 1, 4, and 7) and DA chemotherapy.^22^ In the aforementioned study, GO was also given in consolidation and a high response rate of >90% was reported but some liver toxicity was reported including a case of VOD.

In *NPM1*^mut^ AML, addition of GO to intensive chemotherapy has been reported to increase *NPM1* MRD negativity.^23^ Our results show that the addition of midostaurin further increases this effect. We found enhanced clearance of *NPM1*^mut^ transcripts compared with the cohort of patients with *NPM1*^mut^ receiving DAGO without midostaurin in AML19v1. For patients receiving DAGO+m in AML19v2, 81% were MRD negative in the PB after cycle 2 compared with 68% for DAGO alone in AMLv1. Similarly, the proportion of patients achieving *FLT3* MRD clearance <0.01% was significantly greater in patients receiving DAGO+m compared with DAGO alone.

Regarding the question of the optimal dose of GO, there was no difference in toxicity, and fewer patients receiving the fractionated schedule (DAGO2) tested PB *NPM1* MRD positive after course 2 compared with those receiving DAGO1. This is in keeping with previous observations of a benefit of fractionated GO in reducing MRD in *NPM1*^mut^ AML compared with a single dose.^20^^,^^24^ In AML19v1, the proportion of patients testing PB PC2 MRD negative increased from 69% with DAGO1 to 84% with DAGO2. As we have recently reported that the benefit of transplant in *NPM1*^mut^ AML is confined to those testing MRD positive at this time point, this represents a substantial diminution in the proportion of patients recommended for allo-SCT in first remission^10^; indeed fewer patients receiving DAGO2 in this study received a transplant in CR1. Of note, we recently reported that patients treated with DAGO2 in the AML18 trial had improved posttransplant survival compared with those treated with DAGO1.^20^

It is difficult to compare our results with those reported with for induction chemotherapy with an FLT3 inhibitor but without GO due to differences in the patient populations enrolled. Crudely, the 2-year OS of 78% reported here compares favorably with the reported 2-year OS of 62% for DA plus midostaurin in the RATIFY study^1^ (which included more adverse-risk patients but was limited to those aged <60 years) and 55% for DA plus quizartinib in the QUANTUM-First study^2^ (which was limited to patients with *FLT3*-ITD but included older patients). We observed particularly encouraging results in subgroups including in patients with CBF translocations or *NPM1* mutation (with 2-year OS of 100% and 88%, respectively) and in those aged >60 years, among whom there was no evidence of increased toxicity. Of note, maintenance was generally well tolerated and of 20 patients with molecular MRD markers (17 with *NPM1*^mut^ and 3 with CBF) who completed 12 cycles, all were persistently MRD negative and only 1 patient has relapsed after stopping midostaurin.

A series of trials have suggested that intensification of induction chemotherapy can improve outcomes in *FLT3*^mut^ AML. Both escalated daunorubicin dose of 90 mg/m^2^ in the NCRI AML17 trial^25^ and FLAG-Ida–GO in the NCRI AML19v1 trial^8^ improved OS compared with DAGO. Excellent results have also been reported in *FLT3*mut AML with the combination of CLIA (cladribine, idarubicin, and cytarabine) combined with sorafenib.^26^ Combining venetoclax with chemotherapy and an *FLT3* inhibitor is also being explored (ClinicalTrials.gov identifier: NCT03661307).

These observations clearly warrant the development of randomized studies to definitively assess the effect of GO and intensified chemotherapy regimens when combined with an FLT3 inhibitor. Given our current results, we therefore plan to perform a randomized comparison of DA plus midostaurin, DAGO2+m, and FLAG-Ida–GO plus midostaurin in the recently opened OPTIMISE-*FLT3* trial (ISRCTN 34016918).

Conflict-of-interest disclosure: R.D. reports research support from AbbVie, Amgen, Jazz, and Pfizer; travel support from Servier and Jazz; consultancy with AbbVie, Astellas, Jazz, Pfizer, and Servier; and membership on a data safety and monitoring board with AvenCell Theapeutics. S.K. reports research support from Novartis; travel support from Servier; and consultancy with AbbVie, Astellas, Jazz, Novartis, Pfizer, and Servier. The remaining authors declare no competing financial interests.
