# Supplementary material for: Safety and efficacy of combining midostaurin and gemtuzumab ozogamicin with induction chemotherapy in FLT3-mutated AML
Source: Blood Adv. 2025 Oct 3;9(24):6455–66. doi: 10.1182/bloodadvances.2025017244 (PMC12757529; doi:10.1182/bloodadvances.2025017244)

Safety and Efficacy of Combining Midostaurin and Gemtuzumab Ozogamicin with Induction Chemotherapy in *FLT3* mutated AML: Results From the UK NCRI AML19 Trial.

Supplementary Material

Table of Contents

AML19 list of sites and investigators 2

Supplemental methods – MRD assays 4

Supplementary Table 1 – Baseline characteristics of patients in *NPM1* MRD analysis 6

Supplementary Table 2 – Baseline characteristics of patients in *FLT3* MRD analysis 7

Supplementary Figure 1 – Trial Schema 8

Supplementary Figure 2 – Non-haematological Toxicity of DAGO1+m vs DAGO2+m 9

Supplementary Figure 3 – *FLT3*-ITD MRD in the bone marrow stratified by a) midostaurin exposure across AML19v2 and b) by GO schedule in the MidoTarg sub-study. 10

Supplementary Figure 4 – CIR and RFS – overall and stratified by GO dose 11

Supplementary Figure 5 – Survival of high risk patients undergoing transplant in CR1 following induction with DAGO1+m and DAGO2+m 12

AML19 list of sites and investigators

Aalborg Hospital: Maria Kallenbach, Marianne Severinsen; Aarhus Hospital: Ingolf Molle, Mette Holm, Jan Maxwell Norgaard, Hans Beier Ommen; Aberdeen Royal Infirmary: Dominic Culligan; Addenbrooke's University Hospital: Jenny Craig, Charles Crawley, Pramila Krishnamurthy; Aintree Hospital: Vikram Singh, Walid Sadik, Jeffery Smith; Arrowe Park: Ranjit Dasgupta; Auckland City Hospital: Timothy Hawkins, Claire Hemmaway, Leanne Berkhan, Peter Bowett, Richard Doocey; Basingstoke and North Hampshire Hospital: Alison Milne, Ashok Roy, Sylwia Simpson, Henna Wong; Belfast City Hospital: Claire Arnold, Robert Cuthbert, Damian Finnegan, Mary Francis McMullin

Birmingham Heartlands Hospital: Manos Nikolousis, Richard Lovell, Shankara Paneesha; Blackpool Victoria Infirmary: Paul Cahalin; Bradford Royal Infirmary: Anshu Garg, Sam Ackroyd, Adrian Wiliams; Bristol Haematology and Oncology Centre: Priyanka Mehta; Castle Hill Hospital: Sahra Ali, Andrew Fletcher; Cheltenham General Hospital: Adam Rye, Richard Lush; Chesterfield Royal Hospital: Emma Welch, Robert Cutting; Christchurch Hospital: Ruth Spearing, Liam Fernyhough, Peter Ganly, Steve Gibbons, Mark Smith; Churchill Hospital, Oxford: Paresh Vyas; Clatterbridge Cancer Centre/The Royal Liverpool Hospital: Rahuman Salim, Richard Clarke, Amit Patel, Kathrine Lindsay; Countess of Chester Hospital: Salah Tuegar , Gillian Brearton; Crosshouse: William Gordon, Fiona Elliot, Lisa Kean; Croydon: Fathi Al-Jehani, Shreyans Gandhi, Anna Cowley; Derriford Hospital: Patrick Medd, Hannah Hunter, Tim Nokes, Simon Rule; Doncaster Royal Infirmary: Joe Joseph; Dunedin Hospital: Lucy Pemberton, Annette Neylon; Eastbourne District General Hospital & Conquest Hospital: Richard Grace; Forth Valley: Hugh Edwards, Roderick Neilson, Katrina Farrell, Katharine Hanlon; Freeman Hospital: Gail Jones; Glan Clwyd Hospital: Margaret Goodrick, Earnest Heartin, Christine Hoyle; Gloucestershire Royal Hospital: Adam Rye; Guys Hospital: Kavita Raj, Richard Dillon; Herlev Hospital: Claudia Schollkopf; Hillingdon Hospital: Richard Kaczmarski, Taku Sugai; Ipswich Hospital: Mahesh Prahladan, Debo Ademokun, Isobel Chalmers, Andrew Hodson; James Cook University Hospital: Mohsen Norouzi; James Paget Hospital: Thomas McKerrell, Manzoor Mangi, Shala Sadullah; Kettering General hospital: Mark Kwan; Leicester Royal Infirmary: Kate Hodgson, Ann Hunter, Natalie Garner, Murray Martin; Lincoln County Hospital: Kandeepan Saravanamuttu, Charlotte Kallmeyer, Gamal Sidra, Annette Hilldrith; Manchester Royal Infirmary: Eleni Tholouli , Rachael Crayton, Muhammad Saif; Medway Maritime Hospital: Handunneththi Mendis, Lianwea Chia, Maadh Aldouri; Milton Keynes Hospital: Moez Dungarwalla, Denise White; Monklands Hospital: Pamela Paterson, Jane Clark, Lindsey Mitchell, John Murphy, Alaeddin Raafat; Musgrove Park Hospital: Simon Bolam, Deepak Mannari, Simon Davies, Belinda Austen, Sarah Allford, Sudhakar Kakalamudi; New Cross Hospital: Richard Whitmill, Supratik Basu, Lucy Lynn; New Victoria Hospital: Anne Morrison, Alison McCaig; Ninewells Hospital and Medical Centre: Sudhir Tauro, Keith Gelly, Ron Kerr, David Meiklejohn; Norfolk and Norwich University Hospitals: Angela Collins, Matthew Lawes, Adele Cooper; Northampton General Hospital: Jane Parker, Angela Bowen, Andrea Jones, Catherine Wilde; Nottingham University Hospitals NHS Trust: Nigel Russell, Jenny Byrne, Julie Kenny, Melissa Shaw; Odense Hospital: Claus Marcher; Palmerston North: Bart Baker, Susan Newland; Pinderfield General Hospital: Paul Moreton, William Wong, David Wright, Louise Parker; Poole Hospital: Darshayani Furby, Rebecca Maddams, Louise Heckford; Queen Alexandra Hospital, Portsmouth: Robert Corser, Tanya Cranfield, Mary Ganczakowski, Mary Wands; Queen Elizabeth Hospital, Birmingham: Charles Craddock, Hayley Ellis, Samantha Hughes; Queens Hospital, Romford: Paul Greaves, Kristen Hunt, Karen Fielder; Raigmore Hospital: Caroline Duncan, Joanne Craig, Peter Forsyth; Rigshospitalet: Ove Juul Nielsen, Ole Wei Bjerrum, Peter Kampmann, Lars Kjeldsen, Carsten Niemann; Roskilde: Morten Krogh Jensen, Peter Møller, Jane Damm; Rotherham General Hospital: Arun Alfred, Julie Ball, Dawn Collier; Royal Berkshire: Rebecca Sampson, Henri Groch, Stuart Mucklow; Royal Bournemouth Hospital: Joseph Chacko, Rachel Hall, Renata Walewska; Royal Cornwall Hospital: Bryson Pottinger, Richard Noble, Darren Beech; Royal Derby Hospital: Ian Amott, Julie Edmonds, Adrian Smith; Royal Devon and Exeter Hospital: Jackie Ruel, Paul Kerr, Loretta Ngu, Claudius Rudin; Royal Hallamshire Hospital: Muhammed Moshin, Harpreet Kaur, Kelly Brown; Royal Stoke Hospital: Srinvas Pillai, Paul Ferguson, Peter

Dyer, Deepak Chandra; Royal Surrey: Elisabeth Grey-Davies, Johannes DeVos, Louise Hendry; Royal United Hospitals Bath NHS Foundation Trust: Christopher Knechtli, Josephine Crowe, Sarah Wexler; Russells Hall Hospital: Craig Taylor, Savio Fernandes, Jeff Neilson; Salford Royal Hospital: Rowena Thomas-Dewing, Lorna Milne, Clare Barnes; Salisbury Hospital NHS Foundation: Jonathan Cullis, Calire Smith, Vicky King; Sandwell Hospital: Yasmin Hasan, Richard Murrin, Farooq Wandoo; Singleton Hospital: Unmesh Mohite, Karen Chesters, Julie Turner; Southampton University Hospital: Rowena Thomas-Dewing, Matthew Jenner, Kim Orchard, Deborah Richardson; St Bartholomew's Hospital: Jamie Cavenegh, Kayleigh McCloskey, Heather Oatevee; St George's Hospital: Matt Klammer, Kate Xu Chin, Fenella Willis, Amit Sud; St Helens Hospital: Dat Nicholson, David Taylor, Sally Evans; St James University Hospital: Richard Kelly, David Bowen; St Richards: Santosh Narat, Sarah Janes; Stoke Mandeville Hospital: Renu Riat, Helen Eagleton, Liane Simons; Sunderland Royal Hospital: Victoria Hervey, Shikha Chattret, Annette Nicolle; The Beatson WOS Cancer Centre: Mhairi Copland, Maria Vavyla, Jordan Burgess; The Christie: Mike Dennis, Tim Somervaille, Sven Somerfield, Mohd Mamat, Jo Tomlins, Sana Salesh; The James Cook University Hospital: Marianna David Cotterell, Jamie Maddox, Diane Plews, Ray Dang; The Royal Marsden: David Taussig, Mike Potter, Lauren Ellis, Emma Nicholson, Sally Keat, Thubeena Manichavasagar, Ahmed Alhassani; The Royal Oldham Hospital: Allameddine Allameddine, Farahn Anjum, Poulami Chatterjee, Atanas Stanchev, Jayne Peters, Sarah Lindsay-Holmes, Senthil Chodhouri; Torbay District General Hospital: Deborah Turner, Heather Eve, Steve Smith, Patrick Roberts, Nicholas Rymes, Rui Zhao; University College London Hospitals: Panagiotis Koitaridis, Kirit Ardeshna, Piers Blombery, Asim Khwaja, David Linch, Rakesh Popat, Andres Virchis, Kwee Yong; University Hospital Ayr: William Gordon, Paul Cannon, Fiona Cutler; University Hospital Coventry: Ben Baliff, Beth Harrison, Syed Bokhari, Anton Borg, Oliver Chapman, Nicholas Jackson, Shailesh Jobanputra, Mekkali Narayanan, Peter Rose; University Hospital Crosshouse: Hajer Oun, Matthew Powell, Julie Gilles, Alison Laing, Ian Devanny, Gillian Horne, Lea Haskins, Paul Micallef-Eynaud, Peter Maclean; University Hospital Lewisham: Sunil Gupta, Eti Omoregie, Aarti Sham; University Hospital of Wales: Caroline Alvares, Jonathan Kell, Steve Knapper; Victoria Hospital, NHS FIFE: Kerri Davidson, Lorna McLintock, Stephen Rogers, Peter Williamson; Victoria Hospital: Kerri Davidson, Hazel Cree; Waikato Hospital: Gillian Corbett, Hugh Goodman, Shahid Islam, Humphrey Pullon; Western General Hospital: Victoria Campbell, Peter Johnson; Worcestershire Royal Hospital: Salim Shafeek, Elizabeth Maughan, Juliet Mills; Worthing Hospital: Santosh Narat, John Laurie, George Double; Wycombe Hospital: Reni Riat, Helen Eagleton, Robin Aitchison; York Hospital: Manish Jain, Lee Bond, Laura Munro; Ysbyty Gwynedd: Jim Seale, David Edwards.

Supplemental methods – MRD assays

NPM1 and fusion gene MRD was assessed by RT-qPCR at a central reference laboratory, after each course of therapy and then every 3 months for 2 years with investigators informed of the results. The sensitivity of this assay varies according to sample quality and mutant expression level and is generally above 1×10^{-5}. MRD-positivity was defined as amplification of mutation-specific transcripts before PCR cycle 40 in at least 2 of 3 triplicates as previously described.

FLT3-ITD NGS MRD

FLT3-ITD MRD by next-generation sequencing was performed retrospectively using stored samples with sufficient quantities of genomic DNA as follows.

NGS library preparation

500 ng of genomic DNA input per sample (diluted to 10 µL) was subjected to PCR amplification of *FLT3* exons 14 and 15 using NEBNext® Ultra™ II Q5® 2 x Master Mix (New England Biolabs, MA, USA). This primary PCR included an initial denaturation step (94° C for 2 minutes), eight amplification cycles (denaturation 94° C for 30 seconds, annealing 60° C for 30 seconds, elongation 72° C for 60 seconds) and a final elongation step (72° C for 10 minutes). Products from the primary PCR were cleaned up using AMPure XP beads (Beckman-Coulter Inc, CT, USA) with a ratio of 0.9:1 beads to PCR product. A secondary PCR was performed to incorporate unique dual indexes (Integrated DNA Technologies, IA, USA) using the same reagents and parameters described above except 20 amplification cycles were performed. The final library products were then subjected to another clean-up using AMPure XP beads.

Sequencing

Libraries from 96 samples were pooled for sequencing on a NextSeq 2000 instrument using 600-cycle P1 reagents (Illumina, San Diego, CA). 30% PhiX was used.

Bioinformatics and MRD reporting

Demultiplexed sequencing data was analysed for *FLT3*-ITD variant using the getITD bioinformatics pipeline (https://github.com/tjblaette/getitd) using previously published (default) settings. A list of all ITD variants associated with their insertion start site, length, sequence and number of unique reads were generated.

In samples where no pathological ITD variant was identified, a minimum total read count of 100,000 was required to assign MRD negativity; those with <100,000 were considered non-evaluable and excluded from analysis. For MRD positive samples, we included pathological ITD variants with a VAF% > 0.001% (no lower limit of coverage required); those with VAF% between 0.0001% - 0.001% were also included if they fulfilled either of the following criteria: (1) ≥ 10 unique reads supporting the ITD sequence or (2) ≥ 3 unique reads with supportive evidence of previously detected, identical variants at diagnosis or at an earlier assessment time point. The final reported VAF is the sum of all identified ITDs for each patient sample.

Supplementary Table 1 – Baseline characteristics of patients in *NPM1* MRD analysis

	**All DAGO** N = 55	**DAGO1** N = 26	**DAGO2** N = 29	**All DAGO-mido** N = 48	**DAGO1-Mido** N = 25	**DAGO2-Mido** N = 23
Median age (range)	53 (21 - 68)	53 (31 - 68)	53 (21 - 62)	52 (44, 60)	55 (44, 61)	52 (42, 58)
Prior haematological disorder	1 (1.8%)	0 (0%)	1 (3.4%)	1 (2.1%)	1 (4.0%)	0 (0%)
Prior chemotherapy or radiotherapy	1 (1.8%)	0 (0%)	1 (3.4%)	1 (2.1%)	1 (4.0%)	0 (0%)
WHO Performance status						
Normal activity	37 (67%)	16 (62%)	21 (72%)	22 (46%)	12 (48%)	10 (43%)
Restricted activity	15 (27%)	8 (31%)	7 (24%)	24 (50%)	12 (48%)	12 (52%)
In bed <50% waking hours	3 (5.5%)	2 (7.7%)	1 (3.4%)	2 (4.2%)	1 (4.0%)	1 (4.3%)
WCC median (IQR)	47 (12, 85)	45 (22, 81)	47 (8, 85)	21 (11, 48)	20 (11, 40)	23 (13, 50)
FLT3-ITD	38 (69%)	16 (62%)	22 (76%)	39 (81%)	21 (84%)	18 (78%)
FLT3-ITD allelic ratio, median (IQR)	0.32 (0.14, 0.70)	0.31 (0.12, 0.92)	0.32 (0.19, 0.59)	0.51 (0.24, 0.80)	0.49 (0.08, 0.71)	0.57 (0.27, 0.83)
FLT3 TKD mutation	20 (36%)	11 (42%)	9 (31%)	13 (27%)	6 (24%)	7 (30%)
Cytogenetic risk						
Normal	43 (78%)	18 (69%)	25 (86%)	33 (69%)	15 (60%)	18 (78%)
Other intermediate	10 (18%)	6 (23%)	4 (14%)	9 (19%)	7 (28%)	2 (8.7%)
Adverse	1 (1.8%)	1 (3.8%)	0 (0%)			
Failed	1 (1.8%)	1 (3.8%)	0 (0%)	6 (13%)	3 (12%)	3 (13%)

Supplementary Table 2 – Baseline characteristics of patients in *FLT3* MRD analysis

	**All DAGO** N = 32	**DAGO1** N = 15	**DAGO2** N = 17	**All DAGO-mido** N = 45	**DAGO1-Mido** N = 22	**DAGO2-Mido** N = 23
Median age (range)	52 (18 - 68)	56 (30 - 68)	49 (18 - 62)	52 (24 - 74)	54 (27 - 74)	52 (24 - 72)
Prior haematological disorder	2 (6.3%)	0 (0%)	2 (12%)	1 (2.2%)	1 (4.5%)	0 (0%)
Prior chemotherapy or radiotherapy	0 (0%)	0 (0%)	0 (0%)	1 (2.2%)	1 (4.5%)	0 (0%)
WHO Performance status						
Normal activity	25 (78%)	12 (80%)	13 (76%)	21 (47%)	10 (45%)	11 (48%)
Restricted activity	5 (16%)	1 (6.7%)	4 (24%)	22 (49%)	11 (50%)	11 (48%)
In bed <50% waking hours	2 (6.3%)	2 (13%)	0 (0%)	2 (4.4%)	1 (4.5%)	1 (4.3%)
WCC median (IQR)	36 (8, 101)	28 (15, 64)	70 (7, 114)	21 (9, 46)	20 (11, 39)	21 (7, 50)
FLT3-ITD allelic ratio, median (IQR)	0.30 (0.16, 0.74)	0.27 (0.13, 0.77)	0.38 (0.21, 0.51)	0.52 (0.23, 0.79)	0.51 (0.09, 0.72)	0.60 (0.26, 0.81)
FLT3 TKD mutation	2 (6.3%)	0 (0%)	2 (12%)	5 (11%)	2 (9.1%)	3 (13%)
NPM1 mutation	23 (72%)	11 (73%)	12 (71%)	40 (89%)	21 (95%)	19 (83%)
Cytogenetic risk						
Core binding factor	2 (6.3%)	2 (13%)	0 (0%)	2 (4.4%)	0 (0%)	2 (8.7%)
Normal	19 (59%)	8 (53%)	11 (65%)	29 (64%)	13 (59%)	16 (70%)
Other intermediate	9 (28%)	4 (27%)	5 (29%)	8 (18%)	6 (27%)	2 (8.7%)
Adverse	1 (3.1%)	1 (6.7%)	0 (0%)	0 (0%)	0 (0%)	0 (0%)
Failed	1 (3.1%)	0 (0%)	1 (5.9%)	6 (13%)	3 (14%)	3 (13%)

Supplementary Figure 1 – Trial Schema

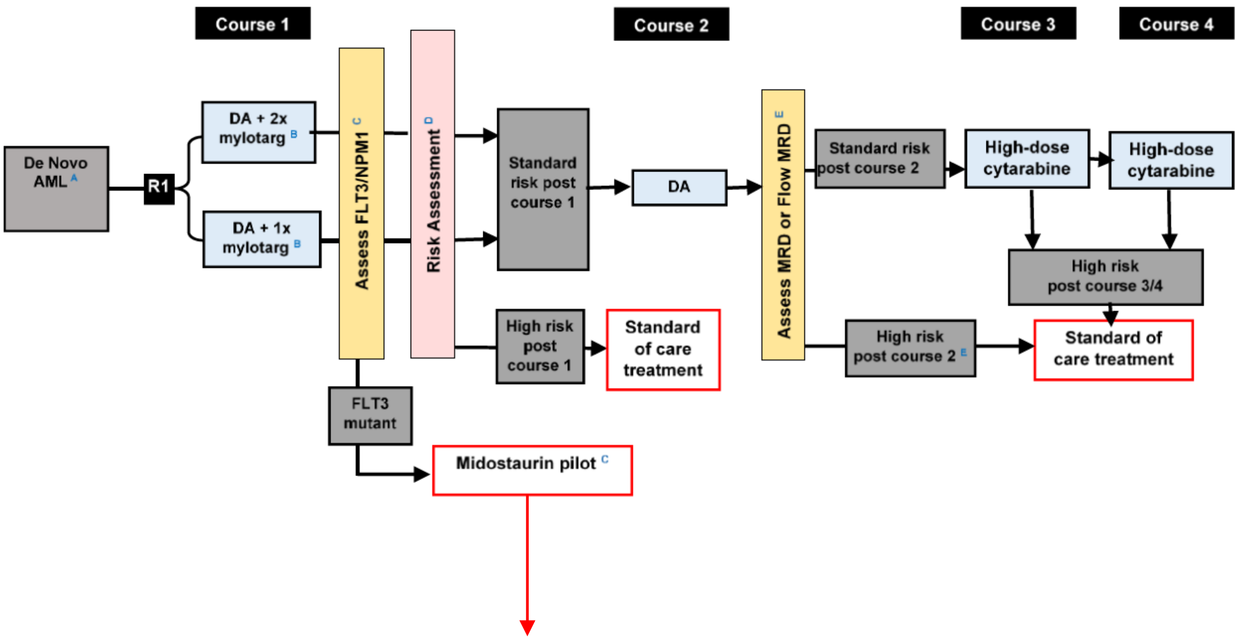

If found to be *FLT3*^{mut} on central testing, patients could enter the Midotarg pilot following separate informed consent, and followed the below schedule.

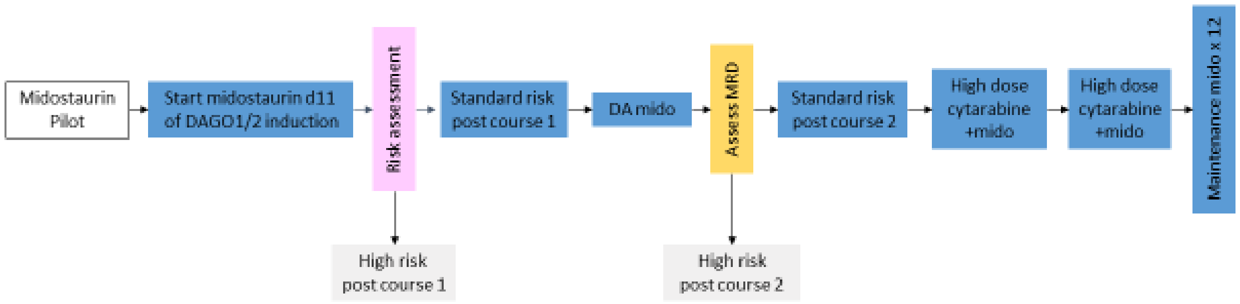

Supplementary Figure 2 – Non-haematological Toxicity of DAGO1+m vs DAGO2+m

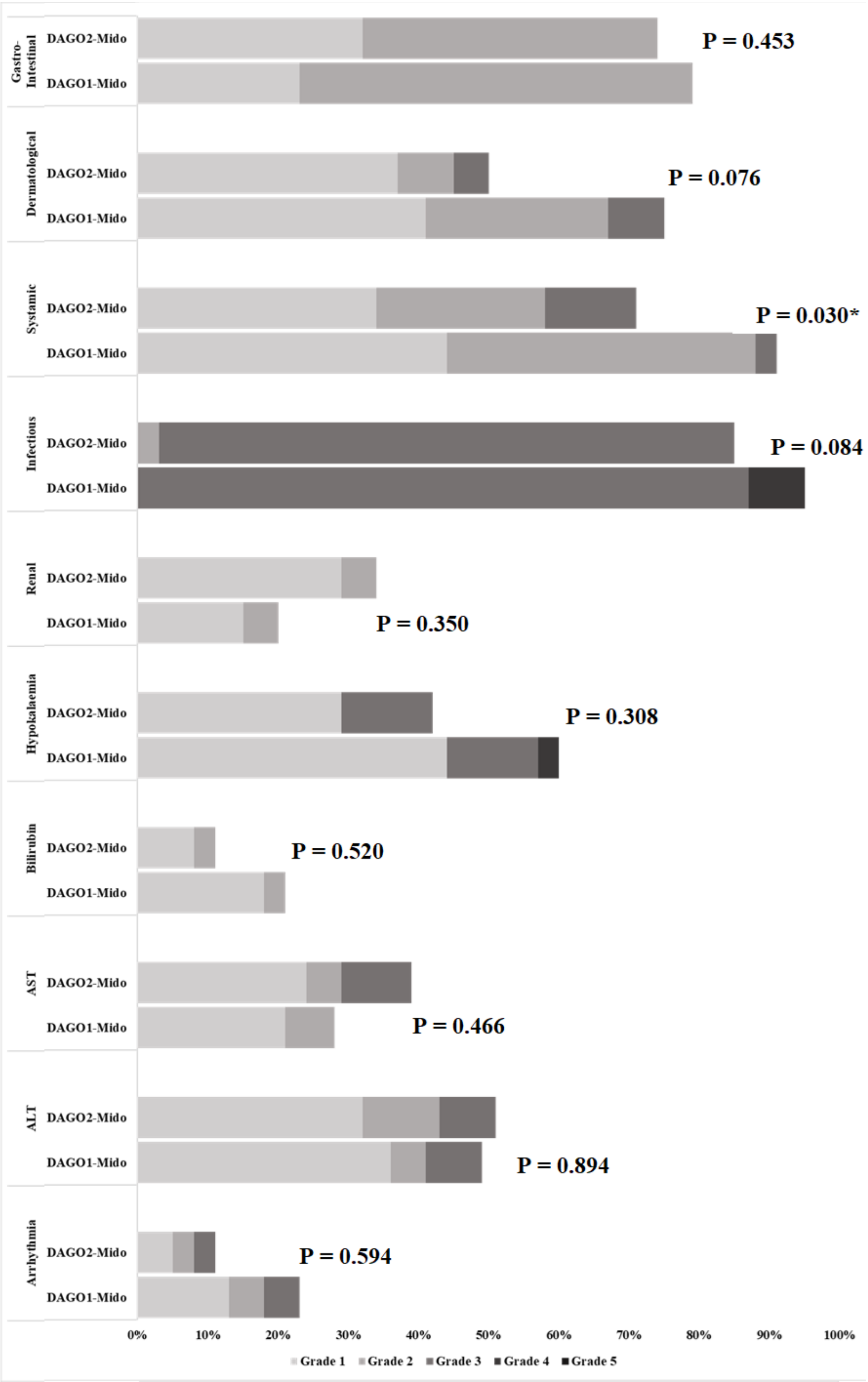

Systemic symptoms comprised weight loss, fatigue, headache and pain

Supplementary Figure 3 – *FLT3*-ITD MRD in the bone marrow stratified by a) midostaurin exposure across AML19v2 and b) by GO schedule in the MidoTarg sub-study.

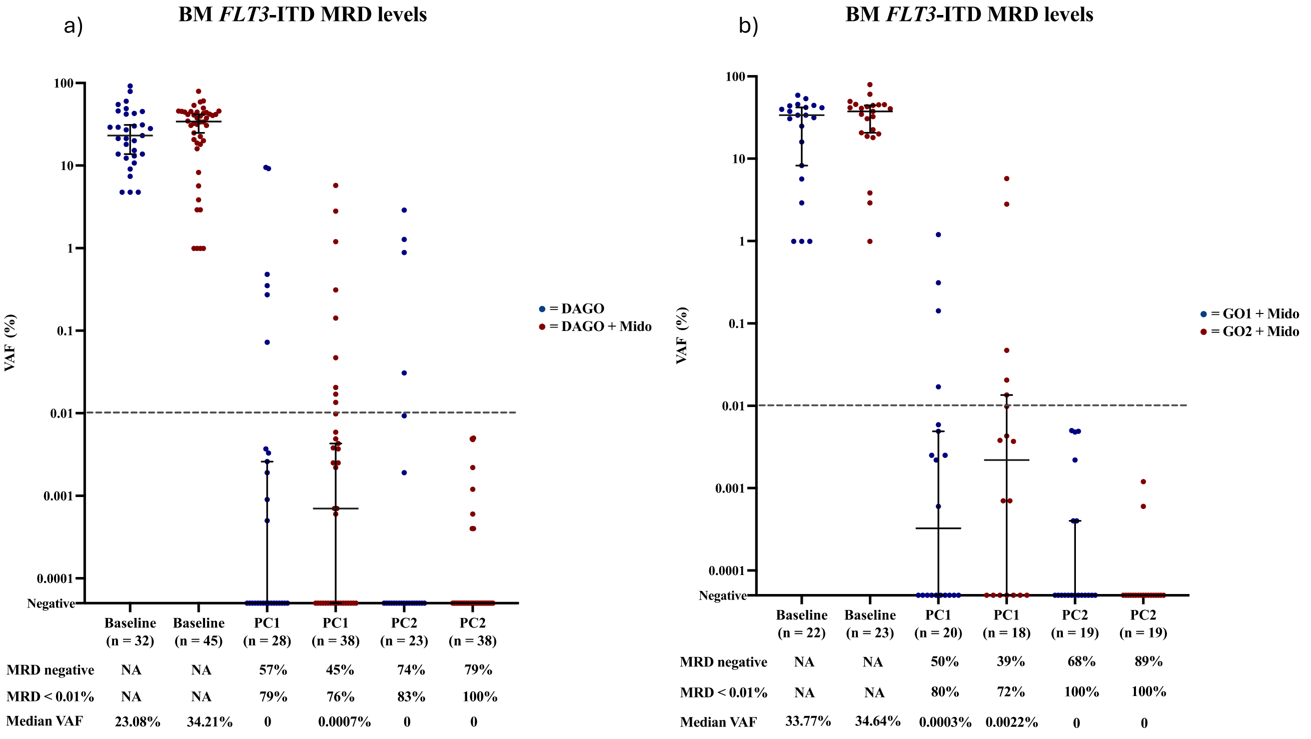

Boxplots depict median and 95% confidence interval. The black dashed horizontal line represents an MRD level threshold of 0.01%.

Supplementary Figure 4 – CIR and RFS – overall and stratified by GO dose

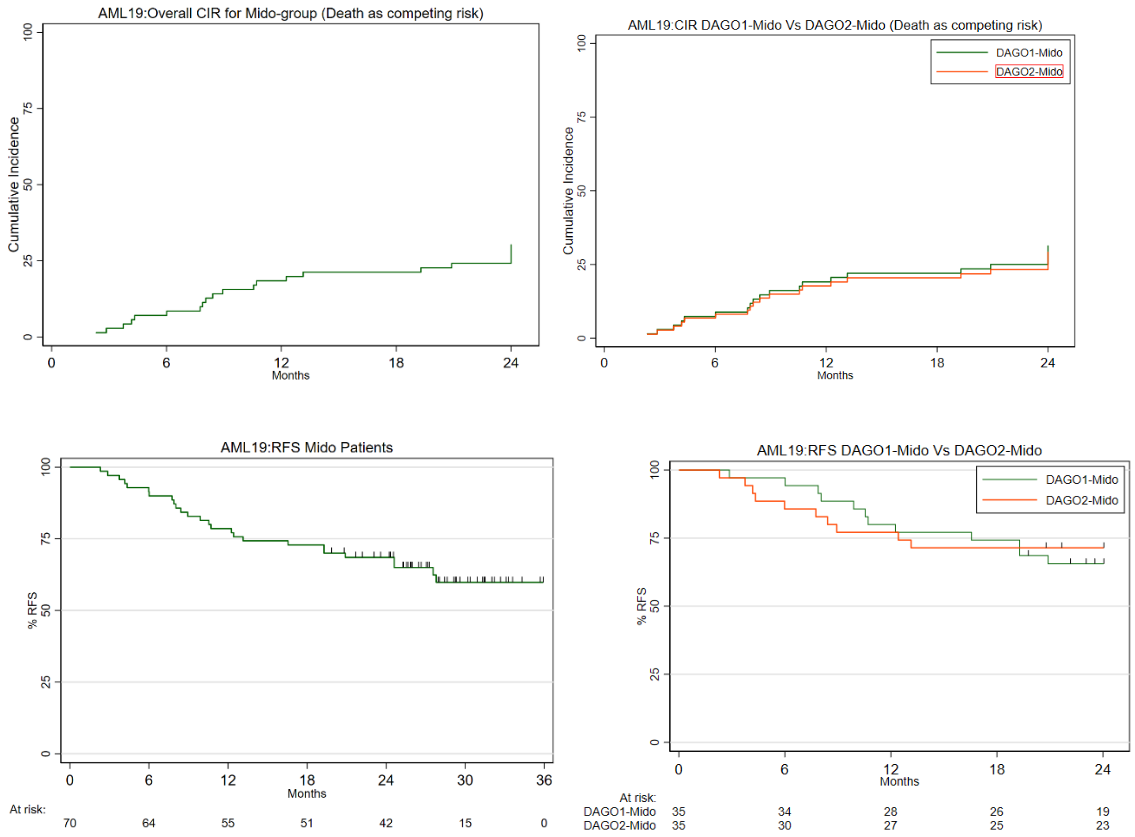

Supplementary Figure 5 – Survival of high risk patients undergoing transplant in CR1 following induction with DAGO1+m and DAGO2+m

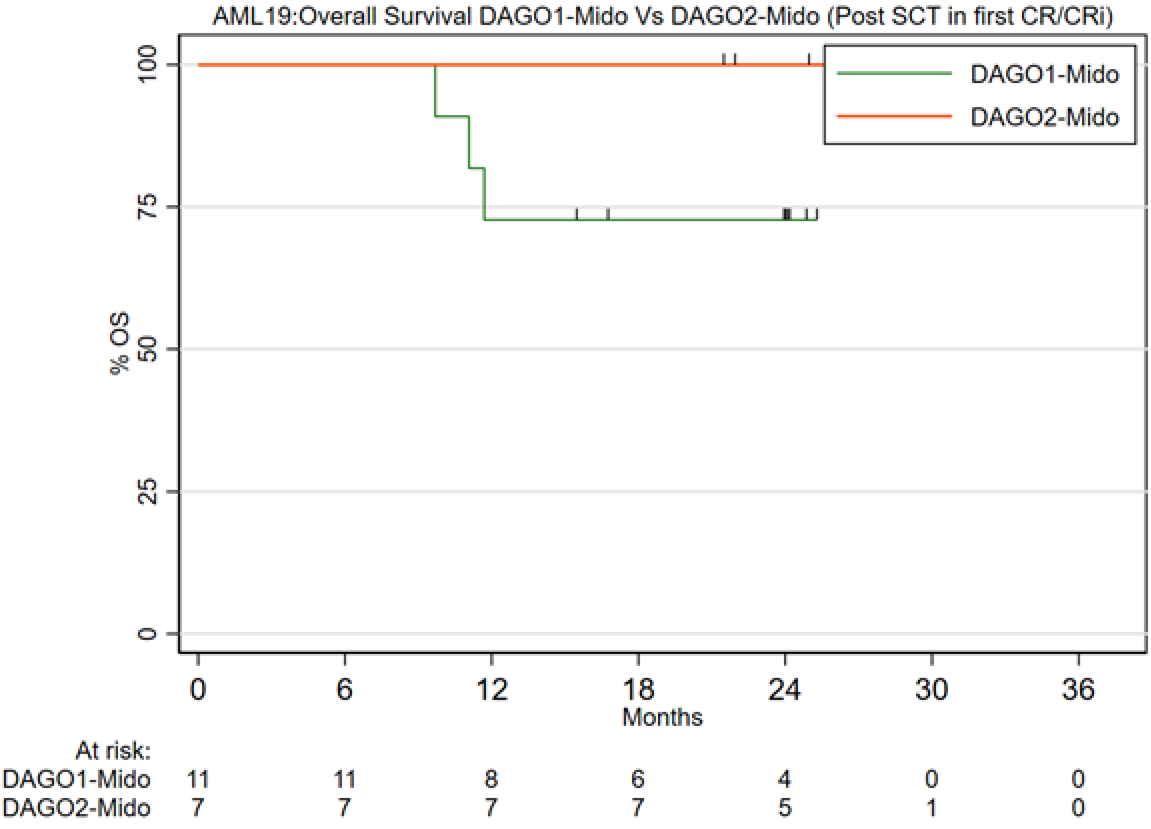

Supplement: Supplemental Appendix, Methods, Tables, and Figures [file BLOODA_ADV-2025-017244-mmc1.pdf]
